# Targeting Epigenetics to Cure HIV-1: Lessons From (and for) Cancer Treatment

**DOI:** 10.3389/fcimb.2021.668637

**Published:** 2021-05-07

**Authors:** J. Peter Svensson

**Affiliations:** Department of Biosciences and Nutrition, Karolinska Institutet (KI), Huddinge, Sweden

**Keywords:** HIV cure, cancer, epigenetics, chromatin, transcription

## Abstract

The Human Immunodeficiency Virus type 1 (HIV-1) integrates in the host genome as a provirus resulting in a long-lived reservoir of infected CD4 cells. As a provirus, HIV-1 has several aspects in common with an oncogene. Both the HIV-1 provirus and oncogenes only cause disease when expressed. A successful cure of both cancer and HIV-1 includes elimination of all cells with potential to regenerate the disease. For over two decades, epigenetic drugs developed against cancer have been used in the HIV-1 field to modulate the state of the proviral chromatin. Cells with an intact HIV-1 provirus exist in three states of infection: productive, inducible latent, and non-inducible latent. Here focus is on HIV-1, transcription control and chromatin structure; how the inducible proviruses are maintained in a chromatin structure that allows reactivation of transcription; and how transcription switches between different stages to allow for an abundance of different transcripts from a single promoter. Recently it was shown that a functional cure of HIV can be achieved by encapsulating all intact HIV-1 proviruses in heterochromatin, giving hope that epigenetic interventions may be used to end the HIV-1 epidemic.

## Introduction

HIV-1 infection increases a person’s risk of acquiring certain types of cancer (Hernández-Ramírez et al., 2017). However, HIV-1 does not tend to cause cancer directly, unlike Human Papilloma Virus (HPV) causing cervical cancer, Hepatitis B or C virus triggering liver cancer, or Human T-lymphotropic virus type-1 (HTLV-1) leading to adult T cell leukemia/lymphoma. Even though HIV-1 is not an oncogenic virus, the HIV-1 integrates in the host genome as a provirus in the reservoir of infected cells and as a provirus HIV-1 shares many features of an oncogene. Both the HIV-1 provirus and oncogenes are harmless in a repressed, latent form and cause disease only when expressed. Successful anticancer therapy necessitates the elimination of all cells with tumor-regenerating potential. Paralleling cancer therapy, to hinder propagation of HIV-1, all the infected cells that can regenerate new infectious HIV-1 particles have to be removed. However, cells with proviruses having incapacitating mutations or those that are permanently silenced may be left untouched (Jiang et al., 2020; Sarabia et al., 2021). Erasing the pathological phenotype can in both cases occur through cell death, or by rendering the cell incapable of producing disease-generating transcripts or proteins. For a functional cure, the therapeutic targets are only the cells able to transcribe intact proviruses or oncogenes.

## Specific Cells to Be Eliminated

Considering the therapeutic target the total reservoir of HIV-infected cells would require a 10,000-fold reduction to achieve a cure according to modelling (Hill et al., 2014). Despite promising *in vitro* results, therapeutic interventions in several clinical trials show no effect to reduce the reservoir or HIV-1 DNA levels (Elliott et al., 2014; Rasmussen et al., 2014; Vibholm et al., 2019). These results cast doubts on the possibility of a complete depletion of the HIV-1 infected cells. The first problem towards an HIV-1 cure is to identify the reservoir of infected cells. On the surface of cells with productive HIV-1 infection, high levels of PD-1, LAG-3, and TIGIT are found (Chomont et al., 2009; Chew et al., 2016; Fromentin et al., 2016; Fromentin et al., 2019). These three surface markers are immune checkpoint molecules, that downregulate the immune response to maintain self-tolerance and prevent hyper-immune activation (Leach et al., 1996; Greenwald et al., 2005). Cancer cells express these surface markers to evade immune response, and immune checkpoint inhibitors (ICIs) are used as anti-cancer immunotherapy (Topalian et al., 2012). The effect of ICIs on the HIV-1 reservoir is unknown but expected to only target the productively infected cells. CD32 is another surface molecule found enriched at productively HIV-infected cells but its function is unclear at this stage (Abdel-Mohsen et al., 2018).

The complexity of the HIV-1 reservoir hinders quantifications of cells with inducible provirus able to generate infectious viruses. The most commonly used PCR-derived methods generally overestimate the actual rebound reservoir. A detailed analysis of the reservoir reveals that most proviruses are defective (Ho et al., 2013; Imamichi et al., 2014; Imamichi et al., 2016; Bruner et al., 2016; Hiener et al., 2017; Pollack et al., 2017; Bruner et al., 2019). Even full length sequencing of intact proviruses overestimates the inducible reservoir as many of these proviruses are permanently silenced (de Verneuil et al., 2018; Jiang et al., 2020; Sarabia et al., 2021). The defective proviruses are unable to propagate HIV-1, even though they are able to produce truncated or chimeric proteins and trigger the immune response (Imamichi et al., 2016; Pollack et al., 2017). However, these cells are still clinically relevant as they exhaust the cytotoxic CD8 T cells. To improve therapy, we need to disentangle the reservoir of cells with defective and intact HIV-1 provirus. In this review, focus is on the reservoir of cells able to generate infectious HIV-1 particles, much like the “cancer stem cells” in a cancer setting.

## The Promise of Repressive Chromatin

A small group of people living with HIV-1–”elite controllers” (ECs)–are able to spontaneously control the infection. The most common mechanism of spontaneous viral control is through specific protective HLA class 1 alleles (International HIV Controllers Study et al., 2010). The EC group also have individuals that control proviral expression (Jiang et al., 2020). This subgroup of ECs has the majority of intact proviruses encoded within heterochromatin regions. Heterochromatin is generally inert, and transitions between chromatin states predominantly occur during differentiation or development. Some ECs are able to long-term control the provirus in heterochromatin, either by targeting the integrants to repressed regions or, more likely, by eliminating the integrants in active regions and retaining the non-expressed proviruses (Jiang et al., 2020). This demonstrates that eliminating the provirus in non-heterochromatin may be a way towards a long-term ART-free remission. If we therapeutically can mimic these ECs by epigenetic silencing of the proviruses, we would achieve a functional HIV-1 cure, according to the “lock-and-block” strategy.

## Proviral Integration Is Guided by Host Proteins

Normally the provirus is not targeted to the silent chromatin regions. The integrations sites have been thoroughly mapped to open chromatin regions (Chen et al., 2017; Battivelli et al., 2018). The integration is guided by the affinity of the viral integrase to host proteins, notably LEDGF, HRP-2, and histones (Shun et al., 2007; Schrijvers et al., 2012; Benleulmi et al., 2017). These host proteins are present at sites of transcriptional activity. HIV-1 provirus integration also occurs in other open structures such as enhancers, possibly by DNA accessibility or other yet undefined factors (Vansant et al., 2020). Within the 3D structure of the nucleus, proviral integration occurs predominantly in the nuclear periphery, within the permissive chromatin regions in proximity to nuclear pores (Dieudonne et al., 2009; Lucic et al., 2019).

## HIV-1 Interacts With DNA Damage Response Pathways

The HIV-1 capsids pass through the nuclear pores intact by interaction with CPSF6, before they are uncoated in the nucleus and integrated in the host genome (Lee et al., 2010; Bejarano et al., 2019; Zila et al., 2021). For the provirus to integrate, a double strand DNA break is required. HIV-1 integration is linked to RAD51 (Thierry et al., 2015), and through the viral Vpr protein, HIV-1 extensively engages several DNA damage response pathways (Li et al., 2020). Vpr plays a key role by inhibiting specific pathways of the DNA repair and also inducing cell cycle arrest and upregulating p21 (Vázquez et al., 2005). Expression of p21 has been found to prevent HIV-1 infection in ECs (Chen et al., 2011). Also by treating macrophages with topoisomerase inhibitors used against cancer, p21 is upregulated which in turn prevents HIV-1 integration (Mlcochova et al., 2018).

## Conditions That Prevent Proviral Expression

**Figure 1:**
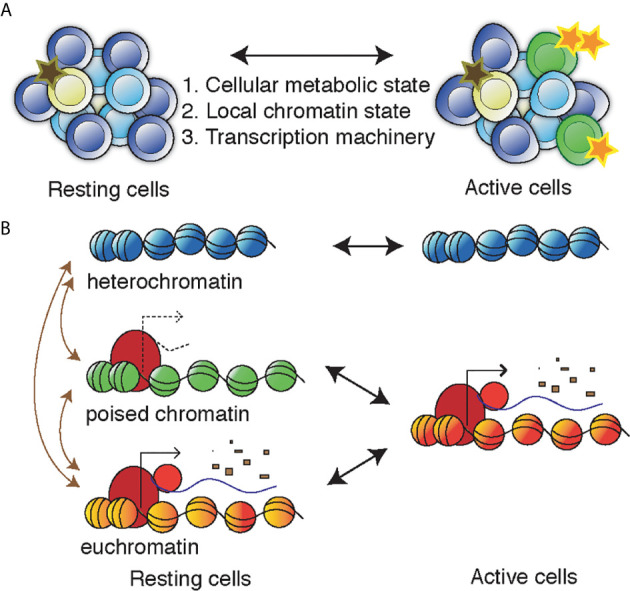
Controlling the proviral/oncogenic activity during T cell activation. **(A)** A heterogenous population of cells can switch between different levels of activity resulting in changes of 1) the metabolic state of the cell, 2) the permissiveness of the chromatin of the provirus/oncogene that can adopt heterochromatin, euchromatin or poised chromatin structures, and 3) the availability of the transcriptional machinery, including transcription factors but also elongation, splicing and polyadenylation factors. **(B)** In the nucleus, the genetic locus of the provirus/oncogene in found in heterochromatin, poised chromatin (non-productive), or euchromatin (productive). Upon cell activation, the poised chromatin can allow productive transcription whereas the heterochromatin remains inactive. Transitioning between chromatin states (maroon arrows) is generally not associated with cell activation, instead it is caused by (de-) differentiation or chromatin resetting and can be induced be epigenetics drugs such as inhibitors of HDACs, BET domains, or DNMTs.

The integration site is a strong predictor of proviral expression, as chromatin modifications at the integration site spread over the proviral sequence (Jordan et al., 2001; Schröder et al., 2002; Lewinski et al., 2005; Brady et al., 2009). Because the HIV-1 virus targets active cells and integration occurs in active chromatin regions, conditions are beneficial for immediate viral production. Analogously to oncogene expression, three conditions need to be met for viral production: metabolically active cells, availability of transcription factors and a permissive chromatin environment (**Figure 1A**). Lack of any these three conditions will lead to proviral latency. As active cells return to quiescence, the metabolism shifts and transcription factors such as NFκB, with multiple binding sites in the HIV-1 promoter, are degraded (Chun et al., 1995; Shan et al., 2017). Even though the provirus is predominantly integrated in open regions, in time the provirus takes on a compact structure, and heterochromatin defining histone modifications appear on the proviral chromatin (Lindqvist et al., 2020). The lysines of histone H3 at position 9 and 27 (H3K9, H3K27) become methylated, representing distinctive heterochromatin structures. These epigenetic marks have been thoroughly described in the context of latent HIV-1 (du Chene et al., 2007; Marban et al., 2007; Pearson et al., 2008; Kauder et al., 2009; Tyagi et al., 2010; Friedman et al., 2011; Tripathy et al., 2015; Bouchat et al., 2016; Kessing et al., 2017; Nguyen et al., 2017; Taura et al., 2019).

## Proviral Latency Is Induced Through Invading Chromatin Marks

Methylated H3K9 serves as a binding platform for heterochromatin protein 1 (HP1), which has three isoforms, α, β and *γ*. HP1α/β are found at compact and inert heterochromatin, mainly in differentiated cells. The observed chromatin compaction in primary cells suggests that HP1α/β accumulate at the provirus (Lindqvist et al., 2020). The HP1*γ* isoform deviates from the other isoforms in that it is found at H3K9me3 chromatin in transcribed regions, often during development. Depleting HP1*γ* in cells from ART treated people living with HIV-1 leads to latency reversal (du Chene et al., 2007). This is one example where the provirus adopts features of undifferentiated cells to adopt a more plastic chromatin structure.

Heterochromatin marks spread across neighboring nucleosomes through e.g., the Human Silencing Hub (HUSH) complex. The HUSH complex is made up of the subunits MPP8 and TASOR. MPP8 recognizes H3K9me3 and TASOR binds to nascent RNA (Douse et al., 2020). In combination, this leads to HUSH being recruited to expressed H3K9me3-marked chromatin. MPP8 in turn recruits the methyltransferase SETDB1 to methylate H3K9 residues of adjacent nucleosomes. In this manner transcriptionally active genetic elements adjacent to heterochromatin are silenced (Tchasovnikarova et al., 2015). Alternatively, Polycomb repressive complex II (PRC2)-mediated H3K27me3 can silence the provirus (Friedman et al., 2011; Kumar et al., 2015; Tripathy et al., 2015; Nguyen et al., 2017). In contrast to constitutively silenced H3K9me3-chromatin, H3K27me3 is found at facultative heterochromatin. PRC2 is frequently mutated or overexpressed in various cancers, including lymphoma and prostate cancer. Small molecules targeting PRC2 are being developed as anticancer therapeutics (Martin et al., 2020). These molecules also have a potential in HIV-1 therapy by preventing heterochromatin from invading the provirus, and thus preventing re-activatable HIV-1 latency.

## Proviral Latency Is Induced by De Novo Silencing

Apart from extending chromatin domains, host mechanisms exist to directly silence foreign elements. Retroviruses, endogenous retroviruses (ERVs), or retrotransposons are targeted by KRAB-ZFP proteins. These small proteins consist of a Zinc finger protein (ZFP) that recognizes specific DNA sequences, and a Krüppel-associated box (KRAB), that recruits a cascade of proteins including SETDB1 to actively silence foreign DNA sequences (Wolf and Goff, 2009; Castro-Diaz et al., 2014; Jacobs et al., 2014; Wolf et al., 2015; Imbeault et al., 2017). A large proportion of the human genome consists of these KRAB-ZFP but no KRAB-ZFP is yet found to recognize HIV-1 sequences.

The role of DNA methylation in silencing HIV-1 is debated (LaMere et al., 2019). Members of the DNA methyltransferase (DNMT) family are often deregulated in cancer and upon HIV-1 infection (Fang et al., 2001; Zhang et al., 2015). The viral Tat protein is able to induce expression of DNMT3 and subsequent DNA hypermethylation (Luzzi et al., 2014). DNMT inhibitors are currently used in the treatment of myelodysplastic syndrome and acute myeloid leukemia. Other regulator of methylation, of both DNA and histones, are the IDH1/2 proteins (Xu et al., 2011). Mutations of these metabolic proteins are frequently found in many types of cancer, and targeted inhibitors of the mutation forms of IDH1/2 are in clinical trials (Raineri and Mellor, 2018). IDH1 has been implicated in HIV-1 infection (Chinn et al., 2010) but its role is yet to be determined. Recently the histone chaperone CAF-1 was found to engage at the long-terminal repeat (LTR) of the HIV-1 provirus. In addition to recruiting chromatin modifiers, CAF-1 may contribute to HIV-1 latency by forming phase-separated nuclear bodies (Ma et al., 2021).

## Maintaining Latency in an Open Chromatin Structure

Other possibilities for HIV provirus to remain non-productive is to block the transcription machinery. The HIV-1 promoter contains regions that have been proposed to form stable G-quadruplexes. These structures prevent transcription and thus regulate promoter activity (Perrone et al., 2013). Also, enhancers are open structures that are transcribed but do not produce mRNAs to be translated. HIV-1 can make use of this strategy to maintain a poised latency (Lindqvist et al., 2020). By being encapsulated in enhancer chromatin (Chen et al., 2017; Battivelli et al., 2018), the HIV-1 provirus maintains a permissive chromatin structure without producing any viral proteins to activate an immune response. Short unspliced enhancer-like transcripts are being produced in latently infected resting CD4 cells (Yukl et al., 2018). The HIV-1 provirus has a splice donor just downstream of the transcription start site, as mammalian genes (Andersson et al., 2014). By ignoring this splice site, the nascent transcripts acquire enhancer transcript features. These persistent transcripts mark the cells responsible for viral rebound as these transcripts predict time to HIV-1 rebound (Moron-Lopez et al., 2019; Pasternak et al., 2020).

## Reservoir Proliferation and Evolution

Maintenance of the reservoir in people living with HIV-1 under ART is driven by cellular proliferation (Kearney et al., 2016; De Scheerder et al., 2019). This proliferation, controlled by host mechanisms, is both homeostatic and antigen-driven (Simonetti et al., 2020). Antigen-driven proliferation can be from HIV-1 antigens, but also from other unrelated antigens. Here the provirus act as a passenger of T cell proliferation. With time under ART, the reservoir evolves and intact proviruses as well as proviruses with an intact 5´ region are lost (Bruner et al., 2019; Pinzone et al., 2019). Possibly these cells express viral proteins toxic to the cell, or they are recognized by CD8 cells. Reservoir cells with a mutated HIV-1 5´ region are more resilient. Through the intact LTR promoter, these cells may express viral proteins encoded in the 3´ provirus, notably Nef, an accessory protein. Nef is able to force internalization of HLA molecules, rendering cells invisible to the immune system. Nef-expressing cells do not decay in time, whereas cells expressing Gag, Pol, Env are eliminated (Thomas et al., 2017; Stevenson et al., 2021). Like in cancer, the HIV-1 reservoir is under evolutionary pressure to evade recognition (Mylvaganam et al., 2019). T cell responses shape the long-term HIV-1 cell population (Akahoshi et al., 2020).

## Poised Chromatin Structures Are Susceptible to HIV-1 Provirus Activation

T cell activation results in increased metabolic activity and activation of a diverse set of transcription factors, including NFκB. Activation of the HIV-1 infected cells correlates with proviral activation. Metabolic activity such as mTOR signaling is required for latency reversal (Besnard et al., 2016). Latency reversal recapitulates the metabolic dysregulation induced during productive infection (Shytaj et al., 2020). Activation of T cells cause changes among the activating chromatin marks such as H3K27ac on the HIV-1 provirus (Lindqvist et al., 2020). However, cellular activation does not generally lead to transition between proviral chromatin states. In contrast to wide-spread chromatin state transition during T cell differentiation where cells shift from naïve to memory T cells, short-term T cell activation does not correlate with global changes in chromatin states (Barski et al., 2009; Cuddapah et al., 2010). Genes inducible by T cell activation are instead associated with poised promotors in resting T cells.

Poised chromatin is defined by the simultaneous presence of active and repressive epigenetic marks, such as post-translational modifications of histones or histone variants. The histone variant H2A.Z is primarily found at promoters but also in coding regions of inducible genes (Sadeghi et al., 2011). H2A.Z is less stable than the canonical H2A and histone turnover will locally allow processive transcription (Svensson et al., 2015). Transcription through chromatin can affect histone turnover depending on the protein complexes associated with RNAPII (Sadeghi et al., 2015). The PAF1 complex has been suggested to evict old histones and thereby make chromatin more accessible to permit transcription of previously non-permissive but poised chromatin. This mechanism could explain the requirement of PAF1 for Tat-mediated HIV transcription (Sobhian et al., 2010).

## Transcription Phases in HIV-1 Proviral Activation

Although accessible proviral chromatin is necessary for engagement of the transcription machinery, it is not sufficient for proviral latency reversal (Battivelli et al., 2018; Sarabia et al., 2021). Upon cellular activation, proviral transcription continue to depend on host mechanisms, through initiation, elongation, splicing and poly-adenylation (Yukl et al., 2018). The transcription cycle of the HIV provirus has several phases. Early in the proviral activation, the nascent HIV-1 transcripts are spliced at multiple positions (Moron-Lopez et al., 2020). These transcripts translate into early viral proteins needed to suppress immune response and upregulate HIV-1 transcription. In later stages of proviral transcription, the splicing machinery is inactivated to produce transcripts encoding late proteins, as well as the full-length HIV-1 genome. The factors inactivating the spliceosome are currently unknown. Whereas spliced transcripts can pose as host mRNA, the full HIV genome and intron-retaining transcripts would be recognized in the cell as foreign or malign. Differential intron retention is detected in almost all cancers (Dvinge and Bradley, 2015), but it is also active in differentiation (Yap et al., 2012; Wong et al., 2013). In CD4 T cells, intron retention is accepted in resting cells, but reduced as the cells activate (Ni et al., 2016). Nuclear export of unspliced HIV-1 transcripts is facilitated by the viral Rev protein, and proximity to the nuclear pore additionally assists in rapid export from the nucleus (Lucic et al., 2019). The oncogene *MYC* uses a similar mechanism to export intron-containing transcripts from nucleus (Scholz et al., 2019).

## Therapeutic Targets for Chromatin State Reversal

A strategy to cure HIV-1 is the “shock and kill” approach, where cells are treated with a latency reversal agent (LRA) to shock the provirus into expression, followed by killing of the cell by either the immune system or a second therapeutic such as broadly neutralizing antibodies (Sok et al., 2016). To stimulate proviral expression in latently infected cells, several drugs developed against cancer have been used as LRAs. Exposing latently HIV-1 infected cells to epigenetic anti-cancer drugs such as inhibitors of HDACs, DNMTs, and combinations thereof stimulates proviral expression (Jiang et al., 2015; Bouchat et al., 2016; Moron-Lopez et al., 2019). These drugs lead to a more open chromatin structure, suggesting a closed chromatin configuration prevents transcription in at least a subset of reservoir cells. Still, however, only a minority of infected cells also reactivate the provirus (Battivelli et al., 2018; Sarabia et al., 2021). An alternative strategy to achieve a functional cure is termed “lock-and-block”. Here the goal is to permanently silence the provirus as to block viral reactivation and prevent HIV-1 transmission in the absence of ART. This strategy has been attempted by e.g. inhibitors of BET or mTOR (Besnard et al., 2016; Niu et al., 2019), or inhibitors of Tat (Kessing et al., 2017).

## Discussion

After long-term ART, intact proviruses endure in infected cells in people living with HIV-1, reminiscent of an oncogene. Even after strong activating signals, proviruses maintain latency. This inertia remains despite nutrients and transcription factors, and predominant integration into open chromatin regions. During initial integration and over time, proviral chromatin adopts different chromatin configurations. As CD4 T cells go from resting to activated, proviruses act according to chromatin state (**Figure 1B**). Proviruses in heterochromatin likely remain in a latent state without producing rebound viruses. Any processive proviral transcription from resting cells is likely to continue during cell activation. Although rare, spliced polyadenylated HIV-1 transcripts have been observed in resting CD4 T cells (Yukl et al., 2018). To remain in long-term circulation, these cells need to deploy mechanisms to actively evade the immune response. For HIV-1 cure purposes, the most interesting reservoir fraction come from cells with the proviruses contained in poised chromatin. Poised chromatin may be associated with unstable promoter histones or being encapsulated in an enhancer-like structure that maintain open accessible chromatin while still avoiding mRNA expression. This fraction of poised proviruses is able to escape immune detection in the resting cell as no viral proteins are expressed, and upon activation produce rebound HIV-1 viruses.

The goal in both cancer therapy and HIV-1 cure is to contain and destroy cells with potential to spread the disease, in a heterogeneous cell population. Both cancer and HIV-1 are evading the immune response. The provirus is using stem cell-like properties and the same pathways as cancer to maintain an open chromatin structure at the provirus. The HIV cure field explores ways in which aberrant transcription occur *via* host mechanisms. Historically HIV-1 has for example been used to decipher the transcription elongation (Kao et al., 1987; Kamine et al., 1996; Mancebo et al., 1997), the role of acetylation (Yamamoto and Horikoshi, 1997; Kiernan et al., 1999; Ott et al., 1999), and promotor remodeling (Verdin et al., 1993; Conrad et al., 2017). The HIV-1 provirus locus continues to be widely used as an appreciated model to understand the host chromatin and transcription machinery of mammalian cells.

## Author Contributions

The author confirms being the sole contributor of this work and has approved it for publication.

## Funding

This work was supported by Vetenskapsrådet (2019-00991), Cancerfonden (19 0412 Pj), Center for Innovative Medicine (FoUI-954473), and Stiftelsen Läkare mot AIDS Forskningsfond (FOb2020-0004).

## Conflict of Interest

The author declares that the research was conducted in the absence of any commercial or financial relationships that could be construed as a potential conflict of interest.
